# Impacts of dietary zinc concentrations on lamb feedlot performance^1^

**DOI:** 10.1093/tas/txaa087

**Published:** 2020-12-22

**Authors:** Ryan M Knuth, Hannah C Cunningham-Hollinger, Berit Bangoura, Alexis L Julian, Chad M Page, Gwendolynn L Hummel, Kelly L Woodruff, Jaelyn R Whaley, Katherine D Bardsley, Scott L Lake, Cody L Gifford, Bledar Bisha, Whitney C Stewart

**Affiliations:** 1 Department of Animal Science, University of Wyoming, Laramie, WY; 2 Wyoming State Veterinary Laboratory, Department of Veterinary Sciences, University of Wyoming, Laramie, WY

## INTRODUCTION

Zinc is one of the most abundant minerals in the body with functions related to immune function and growth performance (Suttle, 2010). However, previous research regarding optimal dietary Zn concentrations has provided varied results observed with average daily gain (ADG), feed efficiency, feed intake, and body weight (BW; Phillips, 1990; Berrie et al., 1995; Fadayifar et al., 2012). Additionally, the effect of a combination of Zn sources (Zn amino acid complex w/ ZnSO_4_) fed at dietary concentrations that meet or exceed NRC requirements in fine-wool feeder lambs has been evaluated to a lesser extent.

Coccidiosis results from a parasitic infection by *Eimeria*, and adverse impacts are mitigated by use of anticoccidial drugs (USDA-APHIS, 2001). During internal development, *Eimeria* spp. multiply inside intestinal cells of lambs. Many host cells are destroyed by *Eimeria* parasites before an oocyst is produced and excreted within the feces, leading to marked intestinal damage (Bangoura and Bardsley, 2020). As a result, small intestine function can be impaired, which affects nutrient utilization and ultimately lamb feedlot performance (Chartier and Paraud, 2012).

Few studies have evaluated the impact of dietary concentration of trace mineral supplementation on coccidia infection in lamb feedlot settings. Provision of trace minerals have decreased intestinal parasite burden in Se supplemented sheep (Hooper et al., 2014) and improved intestinal barrier function in broilers (Zhang et al., 2012) and Crohn’s disease patients supplemented with Zn (Sturniolo et al., 2001), illustrating the role of trace minerals to maintain intestinal health and function. Therefore, strategic Zn supplementation may reduce *Eimeria* oocyst excretion, lessen intestinal damage, and improve overall performance in lambs and optimal levels may differ from recommendations (NRC, 2007). Thus, optimizing Zn concentrations in feedlot diets may be one means of improving lamb performance while simultaneously providing better protection to coccidia infection. The objectives of this study were to identify the impacts of Zn concentrations in a lamb finishing diet on performance and fecal oocyst load. We hypothesized that increasing dietary Zn concentrations would improve lamb feedlot performance and reduce fecal oocyst counts.

## MATERIALS AND METHODS

University of Wyoming (UW) Institutional Animal Care and Use Committee approved all experimental protocols in this project (#20190813WS00386-01).

### Animal Selection and Management

Commercial Rambouillet lambs (*n* = 33; aged 4 mo) of the same sire were selected from the UW flock and stratified by sex and weight into three treatment groups of 11 lambs (43.9 ± 1.0 kg). Lambs were fed a diet fortified with an 80:20 combination of a ZnSO_4_ and Zn amino acid complex (ZnAA; Zinpro Corp.; Eden Prairie, MN, United States) at three different dietary concentrations (treatment, TRT) which met or exceeded current recommendations. Treatments (Table 1) consisted of a corn and soybean hull-based diet with Zn fortified pellet fed at: 1) 1× NRC requirements (450 mg Zn/kg grower pellet; 1NRC), 2) 2× NRC requirements (900 mg Zn/kg grower pellet; 2NRC), and 3) 3× NRC requirements (1,350 mg Zn/kg grower pellet; 3NRC). Each of these values fell below the NRC (2007) maximum tolerable level for sheep when fed at 10% of the diet.

**Table 1.** Dietary and nutrient composition of the finishing diet fed between days 11 and 63

	Treatment^1^		
	1NRC	2NRC	3NRC
Ingredient, %			
Corn	90	90	90
Grower pellet	10	10	10
Nutrient composition, %			
Dry matter	89.89	89.45	89.72
Crude protein	11.5	10.6	11.9
Neutral detergent fiber	20.0	21.2	22.3
Acid detergent fiber	12.8	13.5	15.5
Ash	3.18	2.89	3.72
Mineral composition, %			
Ca	0.50	0.36	0.84
P	0.38	0.36	0.45
K	0.66	0.61	0.80
S	0.15	0.14	0.19
Mg	0.17	0.16	0.19
Na	0.07	0.05	0.15
Fe, mg/kg	84.4	72.8	119.0
Mn, mg/kg	44.0	42.6	84.0
Cu, mg/kg	4.8	4.1	6.0
Zn, mg/kg	72.7	95.5	315.0

^1^Dietary treatments: Zn fortified pellet at 1× NRC requirements (1NRC), 2× NRC requirements (2NRC), and 3× NRC requirements (3NRC).

### Animal Performance

Each group was placed into a dry-lot pen (12 × 5 m) equipped with a GrowSafe feeder (GrowSafe Systems Ltd, Airdrie, AB, Canada) to collect individual feed intake and allowed to acclimate for 10 d. Residual feed intake (RFI) was calculated as the difference between actual and expected feed intake, where actual feed intake was regressed on ADG and metabolic midweight to estimate expected feed intake (Cammack et al., 2005). Start and end BW were measured as a 2-d average on d 0 and 63, respectively, and BW were also recorded on d 14, 28, 42, and 56.

### Fecal Collection and Analysis

Fecal samples were collected on d 0, 14, 28, 42, and 63 following the protocol described by Bauman et al. (2016) and analyzed for parasite stages. Quantification of *Eimeria* spp. oocysts was performed using a modified quantitative McMaster method (Gordon and Whitlock, 1939), and differentiated based on available species descriptions (Vercruysse, 1982; Rommel, 2000; de Macedo et al., 2019).

### Statistical Analyses

The GLIMMIX and analysis of variance procedures of SAS (V. 9.4; SAS Inst. Inc., Cary, NC) were used to analyze feedlot performance traits. The main effects of gain:feed (G:F), ADG, and dry matter intake (DMI) were initially analyzed within TRT, period (d 0 to 11, 11 to 42, and 42 to 63), and TRT × period. Start and end BW and RFI were analyzed within TRT over the entire 63 d trial. Total fecal oocyst counts and counts of major pathogenic *Eimeria* species (*E. ahsata*, *E. bakuensis*, *E. crandallis*, and *E. ovinoidalis*) were log_10_ transformed then analyzed within TRT, day, and TRT × day. Interactions were removed if no statistical tendencies were detected (*P* > 0.10).

## RESULTS

### Feedlot Performance

There was a TRT × period interaction for DMI (*P* < 0.001), but no interaction was detected for ADG or G:F (*P* ≥ 0.15; Table 2). A period effect was observed for ADG and G:F (*P* ≤ 0.04). Lamb ADG and G:F increased by 17% and 29%, respectively, between d 11 to 42 and 42 to 63 across all TRT groups (*P* < 0.05). Start and end BW, ADG, and RFI did not differ by TRT (*P* > 0.46). However, DMI was 12% and 9% greater (*P* = 0.02) for 2NRC and 3NRC compared with 1NRC, respectively.

**Table 2.** Least-squares means for the main effects of zinc dietary concentration and period on RFI (g), study start and end BW (kg), average DMI (kg), ADG (g/d), and G:F

	Zinc dietary concentration^1^			SEM	*P-*value	Period^2^			SEM	*P-*value	TRT × period *P-*value
	1NRC	2NRC	3NRC			d 0–11	d 11–42	d 42–63			
RFI, g^3^	−90.6	73.1	17.5	93.7	0.46	—	—	—	—	—	—
Study start BW, kg^4^	44.12	44.04	43.54	1.68	0.97	—	—	—	—	—	—
Study end BW, kg^5^	64.31	63.79	62.94	2.27	0.91	—	—	—	—	—	—
Average DMI, kg^6^	1.64^b^	1.87^a^	1.81^a^	0.06	0.02	1.67	1.82	1.84	0.06	0.11	< 0.001
ADG, g/d^7^	313.00	310.99	302.26	14.05	0.85	267.05^b^	299.86^b^	359.34^a^	14.05	<0.001	0.97
G:F^8^	0.20	0.17	0.19	0.01	0.36	0.17^b^	0.17^b^	0.22^a^	0.01	0.04	0.15

^1^Dietary treatments: Zn fortified pellet at 1× NRC requirements (1NRC), 2× NRC requirements (2NRC), and 3× NRC requirements (3NRC).

^2^d 0–5: lambs fed a step-up diet consisting of 58% corn, 37% soy hull pellet, and 5% grower pellet; d 5–11: 75% corn, 15% soy hull pellet, and 10% grower pellet; d 11–63: 90% corn and 10% grower pellet (Table 1).

^3^RFI, residual feed intake; calculated as the difference between actual and expected feed intake, where actual feed intake was regressed on ADG and metabolic midtrial BW to estimate expected feed intake then analyzed within Zn dietary concentration.

^4^Study Start BW = 2 d averaged BW measured on d 0.

^5^Study End BW = 2 d averaged BW measured on d 63.

^6^DMI, dry matter intake.

^7^ADG, average daily gain.

^8^G:F, gain:feed.

^a,b^Trait least-squares means within an effect are different (*P* < 0.05).

### Fecal Oocyst Counts

**Figure 1.**
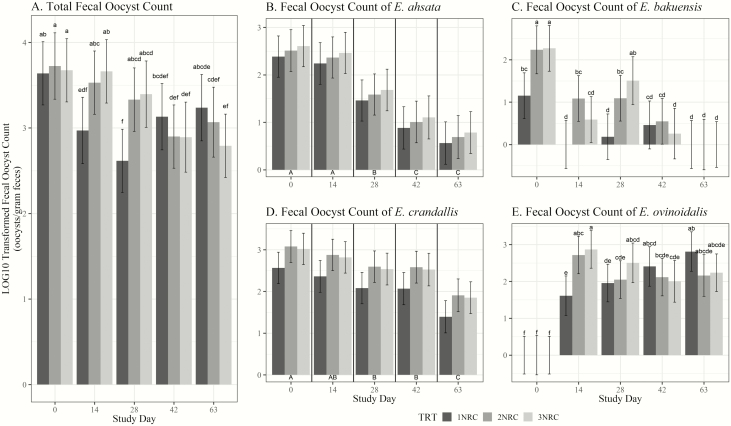
Log_10_ transformed total fecal oocyst count and log_10_ transformed counts of *Eimeria ahsata*, *E. bakuensis*, *E. crandallis*, and *E. ovinoidalis*. (A) Log_10_ transformed total fecal oocyst by Zn treatment (TRT^1^) × day^2^; (B) log_10_ transformed fecal oocyst count of *E. ahsata* by TRT and day; (C) log_10_ transformed fecal oocyst count of *E. bakuensis* by TRT × day; (D) log_10_ transformed fecal oocyst count of *E. crandallis* by TRT and day; (E) log_10_ transformed fecal oocyst count of *E. ovinoidalis* by TRT × day. ^1^Dietary treatments: Zn fortified pellet at 1× NRC requirements (1NRC), 2× NRC requirements (2NRC), and 3× NRC requirements (3NRC). ^2^d 0 to 5: lambs fed a step-up diet consisting of 58% corn, 37% soy hull pellet, and 5% grower pellet; d 5 to 11: 75% corn, 15% soy hull pellet, and 10% grower pellet; d 11 to 63: 90% corn and 10% grower pellet. ^a,b,c,d,e,f^Least-squares means within TRT × day interaction are different. ^A,B,C^Least-squares means within day are different.

A statistical tendency for the TRT × day interaction (Fig. 1) was evident for total fecal oocyst count, *E*. *bakuensis*, and *E. ovinoidalis* (*P* ≤ 0.08). All TRT groups had similar d 0 transformed total fecal oocyst counts, indicating similar initial *Eimeria* load. On d 14 and 28, 1NRC had lower transformed counts than 2NRC and 3NRC by 15% to 23%. Counts of *E. bakuensis* were lower in 1NRC on d 0, 14, and 28 compared with 2NRC and/or 3NRC by at least 48%. Similarly, 1NRC had lower transformed counts of *E. crandallis* on d 14 and 28 than 2NRC and/or 3NRC by 22% to 44%. The effects of TRT were not significant for transformed total fecal oocyst count, *E. ahsata*, or *E. ovinoidalis* (*P* ≥ 0.24). However, TRT affected transformed counts of *E. bakuensis* and *E. crandallis* (*P* ≤ 0.01), where 1NRC lambs had reduced counts for each.

## DISCUSSION

### Lamb Growth

Investigations into the benefits of feeding Zn beyond NRC requirements have shown divergent results (Phillips, 1990; Berrie et al., 1995; Page et al., 2020). Still factorial equations to estimate Zn requirements are dependent on level of production (NRC, 2007; BW, ADG, and wool production) and have not been exhaustively evaluated in U.S. fine-wool sheep when fed an 80:20 combination of a ZnSO_4_ and ZnAA complex. Although DMI was increased with higher Zn inclusion levels, ADG and G:F were unaffected. Still, Page et al. (2020) did not detect any differences in feed intake or efficiency in developing fine-wool rams. These contrasting results may in part be attributed to developing rams fed an alfalfa based diet supplemented with a ZnAA complex or ZnSO_4_ (Page et al., 2020), whereas in the present study feedlot lambs were on a high concentrate ration and supplemented with a combination of the same ZnSO_4_ and ZnAA complex.

### Coccidiosis Incidence and Severity

Few researchers have investigated the impact of dietary Zn concentrations on *Eimeria* spp. In broilers, Bun et al. (2011) reported no effects of dietary organic Zn levels on ADG or DMI. However, effects of organic and inorganic Zn sources on *Eimeria* infections in goat kids have shown reduced oocyst shedding in unsupplemented and Zn oxide supplemented kids compared with Zn chelate and Zn lactate supplemented kids (Strnadová et al., 2011). Of 156 tested fecal samples in the present study, only 4.5% had total fecal oocyst counts less than 100 oocyst per gram (opg) and 11.5% of samples contained more than 10,000 opg, suggesting that coccidiosis spreads easily in an infected population of lambs and can pose a persistent problem. While there was a TRT × day interaction of transformed total fecal oocyst count, *E. bakuensis*, and *E. ovinoidalis*, the impacts of Zn supplementation remain unclear. Even if not all observed reductions of *Eimeria* spp. oocyst excretion were statistically supported, the trend of reduced oocyst counts was obvious for 1NRC compared with 2NRC or 3NRC. Since all *Eimeria* spp. oocysts are products of intracellular parasite multiplication, a decrease in oocyst counts likely reduces intestinal damage and is beneficial for the lamb. Stronger effects may be observed in a setting with occurrence of more severe coccidiosis, and future experimental infection trials may help elucidate the effect of Zn concentrations on *Eimeria* spp.

## IMPLICATIONS

Zinc has many relationships with immune function and animal performance, yet few studies have investigated the impact of dietary Zn concentrations on feedlot lamb performance. The present study has helped fill this void of knowledge and identified current NRC recommendations appear adequate when assessing ADG, G:F, RFI, and BW with DMI being affected. This study was novel to previous studies looking at different dietary Zn concentrations and sources because an 80:20 ZnSO_4_:ZnAA complex was supplemented to fine-wool feeder lambs. Specifically, our data show that DMI was affected by Zn concentration, however no other feedlot performance trait was impacted. Additionally, log_10_ transformed counts of *E. bakuensis* and *E. crandallis* were lower for 1NRC compared with 2NRC and 3NRC lambs. Therefore, these results indicate some benefits of Zn supplementation at 1×, 2×, and 3× NRC recommendations to feedlot lambs, albeit increasing dietary Zn concentrations did not improve many performance measures. Results pertaining to *Eimeria* infections should be interpreted judiciously since they were obtained from a single study in a naturally infected, housed-feeding scenario. Still, future efforts to quantify *Eimeria* spp. response to dietary zinc levels warrant further investigations.



*Conflict of interest statement*. Mention of trade names and commercial products was solely stated to provide specific information and does not imply recommendation or endorsement. The authors confirm that the research was conducted in the absence of commercial or financial relationships that could have influenced the outcome of the study.
